# The cellular roles of Ccr4-NOT in model and pathogenic fungi—implications for fungal virulence

**DOI:** 10.3389/fgene.2013.00302

**Published:** 2013-12-20

**Authors:** John C. Panepinto, Eva Heinz, Ana Traven

**Affiliations:** ^1^Department of Microbiology and Immunology, University at Buffalo, The State University of New YorkBuffalo, NY, USA; ^2^Department of Microbiology, Monash UniversityClayton, VIC, Australia; ^3^Victorian Bioinformatics Consortium, School of Biomedical Sciences, Monash UniversityClayton, VIC, Australia; ^4^Department of Biochemistry and Molecular Biology, Monash UniversityClayton, VIC, Australia

**Keywords:** Ccr4-NOT, fungal pathogen, cell wall, stress adaptation, *Candida albicans*, *Cryptococcus neoformans*

## Abstract

The fungal Ccr4-NOT complex has been implicated in orchestrating gene expression networks that impact on pathways key for virulence in pathogenic species. The activity of Ccr4-NOT regulates cell wall integrity, antifungal drug susceptibility, adaptation to host temperature, and the developmental switches that enable the formation of pathogenic structures, such as filamentous hyphae. Moreover, Ccr4-NOT impacts on DNA repair pathways and genome stability, opening the possibility that this gene regulator could control adaptive responses in pathogens that are driven by chromosomal alterations. Here we provide a synthesis of the cellular roles of the fungal Ccr4-NOT, focusing on pathways important for virulence toward animals. Our review is based on studies in models yeasts *Saccharomyces cerevisiae* and *Schizosaccharomyces pombe*, and two species that cause serious human infections, *Candida albicans* and *Cryptococcus neoformans*. We hypothesize that the activity of Ccr4-NOT could be targeted for future antifungal drug discovery, a proposition supported by the fact that inactivation of the genes encoding subunits of Ccr4-NOT in *C. albicans* and *C. neoformans* reduces virulence in the mouse infection model. We performed bioinformatics analysis to identify similarities and differences between Ccr4-NOT subunits in fungi and animals, and discuss this knowledge in the context of future antifungal strategies.

## Introduction

Model fungal species, such as the yeasts *Saccharomyces cerevisiae* and *Schizosaccharomyces pombe*, have served as powerful genetic models for elucidating the fundamental principles of eukaryotic cellular and molecular biology. In addition to these benign species, the fungal kingdom also contains a number of species that are pathogens of humans, other animals and plants. The pathogenic fungal species have profound effects on the ecosystem, as well as on human health (Brown et al., 2012; Fisher et al., 2012; Kupferschmidt, 2012). In regards to human disease, a small number of species is responsible for most infections, such as *Candida* sp, the mold *Aspergillus fumigatus*, and the basidiomyces yeasts belonging to the *Cryptococcus* species complex (Brown et al., 2012). In most cases, life-threatening disease is seen in hosts with immunocompromised status; therefore fungal infections have become a reason for substantial concern due to the AIDS epidemic and new generation medical procedures that compromise immune function. There are very few treatment options, and mortality from systemic fungal disease remains high in both the developed, and the developing world (Pfaller and Diekema, 2010; Brown et al., 2012). Key mechanisms that impact on the ability of fungi to successfully infect human hosts, survive immune attack, and resist treatments with antifungal drugs include adaptation to growth at host temperature, the ability to mount adaptive responses, genomic and metabolic plasticity, and the capacity to change cell morphology and build and remodel the cell surface, reviewed in Cooney and Klein (2008), Selmecki et al. (2010), Gow and Hube (2012), O'Meara and Alspaugh (2012), Morrow and Fraser (2013). Key regulators have been identified that control these pathogenesis-enabling mechanisms, and many act by modulating gene expression to enable the transitions in cell physiology that favor pathogen survival and virulence. One such regulator is the Ccr4-NOT complex.

Ccr4-NOT is a multisubunit, multifunctional eukaryotic regulator, which has been proposed to serve as a control node for the integration of environmental signals that impinge on cell physiology, as well as acting to coordinate multiple nuclear and cytoplasmic steps in gene expression, reviewed in Collart and Timmers (2004), Collart and Panasenko (2012), Collart et al. (2013). The core Ccr4-NOT complex is composed of nine subunits, which perform roles in gene transcription (initiation and elongation) (Bai et al., 1999; Badarinarayana et al., 2000; Denis et al., 2001; Deluen et al., 2002; Swanson et al., 2003; Qiu et al., 2004; Kruk et al., 2011), posttranscriptional regulation of mRNA stability via poly(A) tail shortening or deadenylation (Tucker et al., 2001, 2002; Chen et al., 2002), as well as several other functions in gene regulation, such as export of mRNAs from the nucleus to the cytoplasm (Kerr et al., 2011), and quality control through interactions with the exosome-dependent pathway (Azzouz et al., 2009a). The architecture is such that the large Not1 subunit serves as a scaffold that brings together the mRNA deadenylases Ccr4 and Caf1 (also known as Pop2) (Bai et al., 1999; Tucker et al., 2001, 2002; Chen et al., 2002), the Not2-5 subunits that mainly function in transcription and contain the Not4 E3 ubiquitin ligase (Collart and Struhl, 1993, 1994; Liu et al., 1998; Bai et al., 1999; Badarinarayana et al., 2000; Hanzawa et al., 2001; Deluen et al., 2002; Panasenko et al., 2006), and Caf40 and Caf130 (Chen et al., 2001). Such complexity of functions in gene regulation, coupled with the fact that Ccr4-NOT activity impacts on the transcriptome in a global way (Cui et al., 2008; Azzouz et al., 2009b; Dagley et al., 2011), means that mutations in the genes encoding Ccr4-NOT subunits lead to pleiotropic phenotypes. In fungi these phenotypes include several that have important implications for virulence of pathogenic species.

Here we provide a synthesis of the knowledge of Ccr4-NOT roles in fungal biology and virulence. We draw on work from *S. cerevisiae*, as the Ccr4-NOT complex has been mostly studied in this model species, and also review what we know about Ccr4-NOT in pathogenic species. We argue that the pleiotropic functions of this complex are beneficial in the context of fungal pathogenesis in two ways: (i) by studying Ccr4-NOT, we can uncover novel mechanistic links between cellular pathways that impact on fungal virulence, and (ii) the pleiotropic functions could be beneficial for targeting the complex with antifungal molecules—inactivating Ccr4-NOT could simultaneously cripple several functions important for virulence, resulting in more efficient elimination of the pathogen. We therefore hypothesize that the Ccr4-NOT complex could be a potentially attractive target for antifungal drug discovery. The Ccr4-NOT complex is conserved throughout eukaryotes, including between yeast and man. This poses a potential problem, as inhibitors of its activity could be toxic to the host. We performed bioinformatics analysis to revisit the conservation between the fungal and animal Ccr4-NOT subunits, and identified several differences that could be exploited for targeting the fungal complex specifically.

## Ccr4-NOT and the fungal cell wall

The fungal cell wall is an essential protective structure built from carbohydrates (glucans and chitin), and glycosylated cell wall proteins. The cell wall mediates interactions that enable attachment of fungi to host cells, adherence to medical implants for the formation of drug resistant biofilms, and immune recognition (Van De Veerdonk et al., 2008; Finkel and Mitchell, 2011; Gow and Hube, 2012). The cell wall is not present on human cells, and as such is an attractive target for antifungal drug therapy. The echinocandin drugs, which are used to treat fungal infections, inhibit cell wall synthesis by acting on the enzyme 1,3 ß glucan synthase (Douglas et al., 1997).

**Figure 1:**
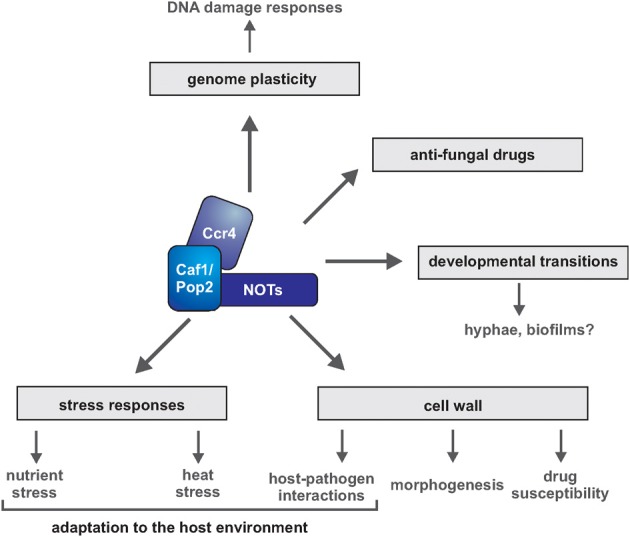
**Multiple roles of Ccr4-NOT in pathways important for virulence of human fungal pathogens**. Gene regulation by the Ccr4-NOT complex has been implicated in multiple survival mechanisms of fungal pathogens, such as cell wall integrity, filamentous growth and survival of nutrient and temperature stress. Direct assessment of the roles of Ccr4-NOT in fungal biofilm formation and host-pathogen interactions is still to come. Subunits of Ccr4-NOT are required for virulence of *C. albicans* and *C. neoformans* in the mouse infection model.

Early work in *S. cerevisiae* has shown that one of the most prominent phenotypes of mutations in Ccr4-NOT relates to defects in the integrity of the cell wall (Betz et al., 2002; Kaeberlein and Guarente, 2002) (Figure 1). Mutants are sensitive to cell wall damaging agents (the echinocandin drug caspofungin, nikkomycin Z that inhibits chitin synthase, calcofluor white, caffeine, SDS), display cell lysis phenotypes, and their growth defects can be suppressed by osmotic stabilization or overexpression of *PKC1* that encodes the kinase of the main fungal cell wall stress response pathway (Betz et al., 2002; Kaeberlein and Guarente, 2002; Markovich et al., 2004; Parsons et al., 2004; Dagley et al., 2011). The cell wall phenotypes are particularly obvious for mutations in the mRNA deadenylase subunits of the complex, *CCR4* and *CAF1/POP2*. Also, inactivation of the exonuclease activity of Ccr4 by mutating one of the catalytic residues mimics a complete deletion of the *CCR4* gene for caspofungin sensitivity (Dagley et al., 2011). The functions of the NOT sub-module and Caf40 and Caf130 are less clear, but there is evidence of involvement. Inactivation of *NOT5* leads to increased levels of expression of genes encoding cell wall functions (Azzouz et al., 2009b), and *not4*, *caf40*, and *not3* mutants are sensitive to caffeine (Parsons et al., 2004; Kapitzky et al., 2010). Mutations in genes encoding proteins that act together with Ccr4-NOT in mRNA turnover, for example the RNA helicase Dhh1 and the RNA binding protein Puf5 (also called Mpt5), also lead to phenotypes consistent with cell wall defects (Moriya and Isono, 1999; Kaeberlein and Guarente, 2002; Dudley et al., 2005; Stewart et al., 2007; Banuelos et al., 2010; Traven et al., 2010; Lo et al., 2012). This argues that mRNA decay of specific transcripts is key for maintaining fungal cell wall integrity.

Ccr4 is also important for cell wall integrity in two prominent human fungal pathogens, *C. albicans* and *C. neoformans*. In these two pathogenic species, *ccr4* mutants display sensitivity to cell wall inhibitors (calcofluor white, congo red, caspofungin), enlarged cells and cell lysis defects (Panepinto et al., 2007; Dagley et al., 2011). In *C. albicans*, similar phenotypes have also been observed for the deletion of *POP2/CAF1* (Dagley et al., 2011). Moreover, the *C. albicans* the *not5* mutant is more sensitive than the wild type to calcofluor white, but more resistant to treatment with the ß-glucanase zymolyase, both phenotypes supportive of alterations in the cell wall (Cheng et al., 2005). *C. albicans* mutants inactivated for *NOT3* display reduced ability to attach to a silicone substrate, which points to changes in cell wall structure and adherence properties (Finkel et al., 2012). In the most direct demonstration roles of Ccr4-NOT in cell wall biogenesis, biochemical analysis of cell wall composition showed that the *C. albicans ccr4* and *caf1*/*pop2* mutants display lower relative levels of cell wall ß-glucans (Dagley et al., 2011). This result is consistent with the sensitivity of the mutants to the echinocandin caspofungin, which inhibits 1,3 ß-glucan synthesis. Given that *S. cerevisiae* and *C. neoformans ccr4* mutants are also sensitive to caspofungin (Markovich et al., 2004; Panepinto et al., 2007; Dagley et al., 2011), it is possible that in those yeasts also Ccr4 impacts on the ß-glucan composition of the cell wall.

**Figure 2:**
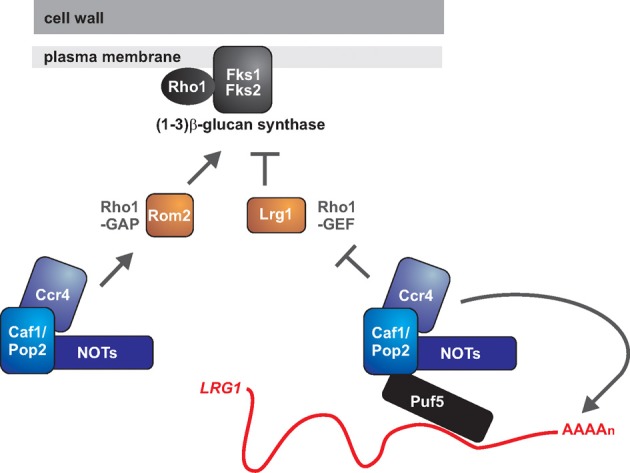
**The roles of Ccr4 in fungal cell wall integrity: is Ccr4 a key regulator of glucan synthesis?** The GTPase Rho1 is the regulatory subunit of 1,3 ß-glucan synthase and might also have a role in the biogenesis of 1,6 ß-glucan. Rho1 is active in its GTP-bound form. The GTPase activating protein (GAP) Lrg1 negatively regulates Rho1, while the GDP exchange factor (GEF) Rom2 is a positive regulator. Ccr4 has been implicated in repressing the expression of *LRG1* and activating the expression of *ROM2*, with both mechanisms acting to promote Rho1 activity. Ccr4 might be repressing *LRG1* via its deadenylase function, and the RNA binding protein Puf5 might be acting to recruit the Ccr4-NOT complex to the *LRG1* mRNA. How Ccr4 acts to promote *ROM2* expression is presently unclear.

What are the target genes controlled by Ccr4-NOT to regulate cell wall biology? Several cell wall-related genes display altered expression levels in *ccr4* mutants, and some of the observed changes could explain the cell wall ß-glucan phenotypes. These data come from the *S. cerevisiae* system. Firstly, in the absence of *CCR4*, *KRE6* encoding a ß 1,6-glucan synthesis gene is expressed at lower levels (Betz et al., 2002). Secondly, Ccr4 might be modulating the activity of ß 1,3-glucan synthase (Figure 2). The levels of two regulators of the GTPase Rho1, which is necessary for ß 1,3-glucan synthase activity, are differentially expressed in *ccr4* mutants (Ito et al., 2011). The GTP exchange factor *ROM2*, an activator for Rho1, is down-regulated, while the GTPase activating protein *LRG1*, a negative regulator of Rho1, is up-regulated. These changes in gene expression likely lead to lower activity of Rho1, which could have a negative impact on ß 1,3-glucan synthase (Figure 2). Rho1 might also have a role in regulating synthesis of ß 1,6-glucans, and is further required for signaling through the PKC-dependent cell wall integrity pathway (Levin, 2011).

The deadenylase activity of Ccr4 negatively regulates gene expression by causing shortening of the mRNA poly(A) tail, thereby initiating decay (Goldstrohm and Wickens, 2008). Therefore, a direct mRNA target of Ccr4 should be up-regulated in the mutant. We would therefore argue that, of the known affected genes, *LRG1* is the most likely direct target of Ccr4 in regards to cell wall phenotypes. Further support for this idea comes from studies of Puf5, an RNA binding protein that binds to 3′ untranslated regions (3′ UTRs) of mRNAs and recruits the Ccr4-NOT complex for deadenylation and translational repression (Goldstrohm et al., 2006). Puf5 also negatively regulates *LRG1* levels (Stewart et al., 2007), and it could be doing so via its interaction with Ccr4-NOT, but this needs to be experimentally demonstrated (Figure 2). Whether *LRG1* is a relevant Ccr4-target gene in other fungal species remains to be studied.

It is very likely that the effects of Ccr4-NOT on the fungal cell wall are complex, and regulation of multiple, functionally distinct genes might be involved. The *C. albicans not3* mutant displays altered levels of several transcripts that encode cell wall proteins (Finkel et al., 2012). Additionally, studies in *C. albicans* have suggested that the effects of Ccr4 on another pathway, mitochondrial biogenesis, might be linked to its functions in cell wall integrity (Dagley et al., 2011). This, and work that followed from it, has revealed that mitochondrial activity in *C. albicans* impacts on cell wall integrity and on the ability to respond to treatment with cell wall inhibitors, including the echinocandin drug caspofungin (Dagley et al., 2011; Qu et al., 2012). These studies provide a case in point for our hypothesis that studying the pleoitropic roles of Ccr4-NOT in fungi can reveal unexpected connections and novel players in cellular pathways important in the context of virulence. Studies in *S. cerevisiae* have revealed respiratory and other mitochondrial defects for several Ccr4-NOT subunits, as well as other factors of the mRNA decay pathway (for example Dhh1) (Betz et al., 2002; Dimmer et al., 2002; Perrone et al., 2005). It is therefore possible that, more generally, Ccr4-NOT and the mRNA turnover machinery link mitochondrial activity with cell wall integrity.

## Ccr4-NOT and the morphogenetic switch

Fungi have the ability to switch between developmental states, which differ in cell morphology, cell surface structure, pathogenicity and interaction with host immunity. One such developmental switch that has been extensively characterized is the yeast to hyphal transition, in which cells change cell morphology from ovoid to elongated filaments (Sudbery, 2011). For *C. albicans*, the hyphal transition is one of the most recognized pathogenic attributes (Sudbery, 2011).

In *S. cerevisiae*, pseudohyphal differentiation in diploid strains and agar invasion in haploid strains are two phenotypes that depend on the morphogenetic switch. In large-scale mutant collection screens, *not5* mutants were found to display enhanced filamentation on the surface of plates but no invasive growth (Jin et al., 2008), and *caf130* mutants were identified as invasion-defective (Ryan et al., 2012). Ccr4 and Pop2 impact on the expression of a key cell surface molecule implicated in invasive and pseudohyphal growth, the adhesin Flo11 (Lo et al., 2012). Ccr4 might be promoting *FLO11* transcription by inhibiting the expression of two transcriptional repressors of *FLO11*, *NRG1* and *NRG2* (Lo et al., 2012). Low Flo11 levels in the deadenylase mutant alter the cell wall properties, and lead to low adherence to polystyrene (Lo et al., 2012). Cells inactivated for the helicase Dhh1 also display similar phenotypes (Park et al., 2006; Lo et al., 2012). Collectively, these results argue that the mRNA decay pathway plays important roles in linking morphogenesis and cell wall regulation.

Studies in *C. albicans* support the *S. cerevisiae* data. Mutations in *CCR4*, *CAF1/POP2*, *NOT1*, *NOT3*, and *NOT5* give rise to cells that are defective in transitioning from yeast to hyphal growth (Cheng et al., 2003; Epp et al., 2010; Dagley et al., 2011). Substrate adherence and hyphal growth are important for the formation of *C. albicans* biofilms (Finkel and Mitchell, 2011). Given that Ccr4-NOT impacts on both, it is possible that this complex controls the ability of *C. albicans* to make biofilms.

The target genes of Ccr4-NOT for the filamentous growth pathway are not known. The biogenesis of the cell wall is intimately linked to filament formation. Therefore, the hyphal defects of the *ccr4-not* mutants could be linked to altered cell wall structure and ß-glucan levels. Consistent with this idea are reports that in *C. albicans* defects in cell wall ß-glucans lead to reduced ability to form hyphae (Lussier et al., 1998; Herrero et al., 2004; Umeyama et al., 2006). It is also possible that Ccr4-NOT has more specific roles in orchestrating the changes in gene expression programs during the developmental switch from yeast to filaments. Transcriptome-wide approaches to study how Ccr4-NOT impacts on gene regulation during the morphogenetic switch in both *S. cerevisiae* and *C. albicans* will answer these questions. Based on current knowledge, we predict that the role of Ccr4-NOT in gene expression is conserved between these two divergent yeasts. However, often, analysis of transcription factor function in different fungal species has revealed a large extent of evolutionary change (Lavoie et al., 2009). How posttranscriptional gene regulation has evolved in fungi is much less understood, and studies of Ccr4-NOT could shed light on this exciting question.

## The ccr4-NOT complex reprograms gene expression to promote stress adaptation

When colonizing the human body, fungal pathogens face numerous stressful conditions, such as nutrient limitation, higher temperature, and low oxygen. In *S. cerevisiae*, *C. neoformans* and *C. albicans* Ccr4-NOT has been implicated in the adaptation to nutrient, temperature and oxygen stress, suggesting this complex is a key player in adaptive response that mediate fungal growth and survival in the host. In this section, we will briefly touch on the *S. cerevisiae* work, as this has been reviewed before (Denis and Chen, 2003; Collart and Timmers, 2004), and focus more prominently on new knowledge gained in the pathogenic species.

Glucose is the preferred carbon source for yeasts, which they ferment by glycolysis. In *S. cerevisiae* the Ccr4-NOT complex has important roles for growth in the absence of glucose (so called non-fermentative growth), reviewed in (Denis and Chen, 2003; Collart and Timmers, 2004). Transcriptome profiling by microarrays confirmed a role for Ccr4-NOT in adaptation to glucose depletion, and also more generally in the response to stress (Cui et al., 2008; Azzouz et al., 2009b). A major effect of Ccr4-NOT on stress responsive gene transcription could be mediated by interactions with the SAGA histone acetyltransferase complex (Cui et al., 2008). Ccr4-NOT has also been implicated in regulating the function of stress-responsive transcription factors, such as Msn2 and Skn7 (Lenssen et al., 2002, 2005, 2007). In the niches in the human body, glucose is scarce, and therefore adaptation to alternative carbon sources is important. It remains to be studied how exactly Ccr4-NOT controls the response to non-fermentative conditions in pathogenic fungi. Transcriptome analysis of the *ccr4*Δ/Δ mutant in *C. albicans* showed that a large proportion of the differentially expressed genes are related to the function of mitochondria (Dagley et al., 2011), suggesting a role in metabolism. Specific effects on gene expression of mitochondrial functions have also been observed in *S. cerevisiae ccr4-not* mutants (Cui et al., 2008). Interestingly, the transcriptional profile of the *ccr4*Δ/Δ mutant of *C. albicans* correlates with the early response to hypoxia, suggesting a role for Ccr4 in low oxygen conditions (Adnan Sellam and André Nantel, personal communication, manuscript in preparation). Hypoxia is commonly encountered by fungal pathogens in the niches in the human body, and the ability to respond to hypoxia is key for virulence (Grahl et al., 2012).

In *C. neoformans*, the Ccr4-NOT complex impacts on the expression of a key virulence factor, the laccase *LAC1*, a multi-copper oxidase required the production of the protective pigment melanin. Work on *C. neoformans VAD1*, the homolog of *S. cerevisiae DHH1*, implicated Not1 as a repressor of *LAC1* gene expression (Panepinto et al., 2005). The *ccr4*Δ mutant is defective in laccase activity, but *LAC1* mRNA induction is not defective (Bloom and Panepinto, unpublished). These data suggest that the defect in laccase production is posttranscriptional. It appears therefore that in *C. neoformans* Ccr4-NOT regulates *LAC1* via Not1-dependent effects of transcription, and Ccr4-dependent posttranscriptional control. The expression of the *LAC1* gene in *C. neoformans* is tightly regulated by glucose repression, suggesting that the role of Ccr4-NOT in glucose-regulated gene expression is conserved between *C. neoformans* and *S. cerevisiae*.

A key stress responsive function of Ccr4 in *C. neoformans* is the response to temperature stress. This is key for virulence. Mammals, such as us, are normally quite resistant to fungal infections, because most fungi do not grow optimally at our body temperature of 37°C. In order to colonize the human body niches, fungal pathogens need to be able to adapt to growth at 37°C. The *C. neoformans ccr4*Δ mutant exhibits temperature sensitivity, and is attenuated in virulence (Panepinto et al., 2007). In response to temperature stress in *S. cerevisiae*, mRNAs that encode ribosomal proteins and other protein synthesis factors are destabilized by Ccr4, suggesting that mRNA decay is a mechanism that promotes stress-responsive re-programming of mRNA pools (Grigull et al., 2004). The destabilization of ribosomal protein mRNAs in response to temperature is also dependent on the RNA polymerase II subunit Rpb4 (Goler-Baron et al., 2008; Harel-Sharvit et al., 2010). The current paradigm is that, under stress conditions, Rbp4 marks transcripts for rapid degradation via co-transcriptional association. In *C. neoformans* the roles of Ccr4 and Rpb4 in the regulation stress responsive changes in mRNA stability is conserved, suggesting that the coupling of mRNA synthesis and decay is likewise conserved (Bloom et al., 2013). In wild type *C. neoformans*, a shift from 30°C to 37°C is accompanied by acute and transient changes in mRNA abundance and stability. Ribosomal protein mRNAs are transiently repressed, and ER stress mRNAs are transiently induced (Havel et al., 2011; Bloom et al., 2013). Between 2 and 3 h post-shift, these mRNAs return to pre-shift levels. In the *ccr4*Δmutant, ribosomal protein mRNAs accumulate and transient repression is absent. Those mRNAs were found to be stable in the *ccr4*Δ mutant, confirming defects in mRNA degradation as the cause of their up-regulation. Likewise, in a *C. neoformans rpb4*Δ mutant, mRNAs encoding ribosomal proteins are stabilized, and the transient repression of ribosomal protein transcript mRNA abundance that accompanies temperature adaptation is attenuated (Bloom et al., 2013). Furthermore, in *ccr4*Δ cells, increased ER stress mRNA abundance persists past 3 h (Havel et al., 2011). The major ER stress sensor is Kar2/BiP, an ER resident Hsp70 family member. In wild type *C. neoformans*, the *KAR2* transcript exhibits distinct changes in mRNA stability in response to temperature stress (Bloom et al., 2013). Under unstressed conditions, the *KAR2* mRNA is very stable, but is destabilized following the shift to 37°C, through Rpb4-dependent and -independent mechanisms, and the data is consistent with a role in mRNA degradation limiting both the intensity and the duration of the stress response. The destabilization of *KAR2* at peak mRNA abundance will prevent persistent activation of the ER stress response such as that seen in the *ccr4*Δ mutant. Phenotypically, there is no change in wild type *C. neoformans* during this adaptation, and growth rate is maintained through the shift. In a *ccr4*Δ mutant, however, growth is nearly arrested, cell morphology becomes aberrantly large and buds fail to separate (Panepinto, unpublished). Clearly, Ccr4 in *C. neoformans* is buffering phenotypic change in response to environmental shift.

## Ccr4-NOT and genome stability: roles in fungal adaptation driven by chromosomal alterations?

Pathogenic fungi can acquire macro and micro-chromosomal alterations that might be beneficial to their survival in the host, or in the face of antifungal drug challenge, reviewed in (Selmecki et al., 2010; Morrow and Fraser, 2013). Cells can be generated that display changes in chromosome numbers (aneuploid cells) or even the whole chromosomal set (ploidy changes). Other chromosomal rearrangements, such as translocations, truncations, gene amplifications and loss of heterozygosity (LOH) are also observed. Pathways that impact on genome stability (DNA replication, damage and repair mechanisms) and cell division (cell cycle control mechanisms) are expected to have an impact on the ability of fungal pathogens to adapt by chromosomal alterations. Work in *S. cerevisiae* and *S. pombe* implicates Ccr4-NOT in the regulation of genome stability. Data in *C. neoformans* is consistent with this function, as *ccr4* mutant cells are sensitive to DNA damaging agents (Panepinto, unpublished).

Mutations in the Ccr4-NOT complex have been repeatedly found in *S. cerevisiae* screens that used the deletion collection to find genes that control susceptibility to DNA damaging agents, as well as by researchers using candidate gene approaches (Bennett et al., 2001; Betz et al., 2002; Hanway et al., 2002; Hartman and Tippery, 2004; Parsons et al., 2004; Westmoreland et al., 2004; Mulder et al., 2005; Traven et al., 2005; Woolstencroft et al., 2006; Gaillard et al., 2009). In *S. pombe*, mutations in Ccr4-NOT result in similar DNA damage response phenotypes (Takahashi et al., 2007; Deshpande et al., 2009). Genetic interactions between genes encoding Ccr4-NOT subunits and genes of the DNA damage checkpoint and repair pathways also support an important role for Ccr4-NOT in DNA damage responses (Traven et al., 2005; Pan et al., 2006; Woolstencroft et al., 2006; Gaillard et al., 2009).

How does Ccr4-NOT activity regulate DNA damage responses? Studies from the *S. cerevisiae* system suggest a complex scenario, with three possible mechanisms that are not mutually exclusive. Firstly, it seems that Ccr4-NOT has a general (non-gene-specific) roles in transcription-coupled repair (TCR) (Gaillard et al., 2009). The TCR roles could be functionally linked to the function of Ccr4-NOT in transcriptional elongation, but the exact mechanism remains to be understood (Gaillard et al., 2009). Secondly, Ccr4-NOT has gene-specific roles in the response to DNA replication stress: Ccr4-NOT is needed for the induction of ribonucleotide reductase (*RNR*) genes, (Mulder et al., 2005; Woolstencroft et al., 2006; Kruk et al., 2011). The effects of Ccr4-NOT on the expression of *RNR* genes are transcriptional and posttranscriptional. Ccr4 might be acting to inhibit the expression of the transcriptional repressor of the *RNR* genes, *CRT1*, by shortening its mRNA poly(A) tail (Woolstencroft et al., 2006). In regards to transcriptional control, in absence of Not4 recruitment of RNA polymerase II and the TATA binding protein (TBP) to the promoter of *RNR3* in response to HU treatment is defective (Mulder et al., 2005). It has been shown more recently that Ccr4 and Not5 are found at the *RNR3* gene in response to MMS, supporting a direct role in transcription of this gene upon DNA damage (Kruk et al., 2011). The third mechanism relates to a co-factor role for Pop2/Caf1 in regulating the subcellular localization of ribonucleotide reductase in response to inhibition of DNA replication (Takahashi et al., 2007). Ccr4 also appears to have some role in this process (Takahashi et al., 2007).

In *C. neoformans*, multiple DNA damage-related mRNAs were found up regulated in the *ccr4*Δ strain by microarray analysis (Havel et al., 2011), suggesting conservation of a role for the mRNA decay arm of the Ccr4-NOT complex in genome integrity. *C. neoformans* lacks an ortholog of *CRT1*, and induces a basidiomycete-specific ribonucleotide reductase small subunit in response to HU treatment, rather than the retained ascomycete-like gene (Zulkifli et al., 2012). An effect of *CCR4* deletion on the regulation of these genes has yet to be determined. Further studies will define the extent of conservation between *C. neoformans* and the ascomycete yeasts.

The implication of the roles of Ccr4-NOT in genome stability is that in the absence of this complex, genome instability should increase, causing higher chromosomal rearrangements. Increased LOH has been shown in the *S. cerevisiae ccr4* and *pop2* mutants, but not for all markers tested, and was most pronounced for a locus on chromosome XII (Andersen et al., 2008). The increased LOH was suggested to be linked to the impact of Ccr4 and Pop2 on maintenance of dNTP pools: chromosome XII contains the rDNA repeat, and it was suggested that the stability of this repeat might be sensitive to nucleotide levels (Andersen et al., 2008). Higher chromosomal instability could favor adaptation of pathogens, such as *C. albicans* and *C. neoformans* to the host environment and antifungal drug stress. Interesting in this context is a recent report that suggests that in *S. pombe* and *S. cerevisiae*, Ccr4-NOT is necessary for ensuring survival of cells that contain chromosomal aneuploidies (Tange et al., 2012). Mutants in the *NOT2*, *NOT3*, and *CAF40* genes were particularly affected. The deadenylase subunits *CCR4* and *POP2* also likely have a role, but this could not be precisely determined due to non-specific effects of deadenylase activation in the assays used (Tange et al., 2012). Bone-fide DNA repair genes were also found to be required for growth of aneuploid cells, and it was suggested that the DNA damage response functions of Ccr4-NOT are linked with the roles of the complex in survival of aneuploids (Tange et al., 2012). These results suggest that some forms of chromosomal rearrangements, such as changes to copy numbers, would not be tolerated in the absence of Ccr4-NOT. This could be important in the context of fungal virulence and antifungal drug tolerance, as aneuplodies are observed frequently, and have been shown in some cases to confer increased resistance to drug treatments, reviewed in Selmecki et al. (2010), Morrow and Fraser (2013).

## Would ccr4-NOT be an appropriate target for antifungal drug development?

The phenotypes of the mutants argue yes. As discussed above, inactivation of Ccr4-NOT function cripples several pathways important for fungal virulence. Moreover, inactivation of *CCR4* in *C. albicans* and *C. neoformans*, as well as *NOT5* in *C. albicans* resulted in significantly crippled virulence in mice (Cheng et al., 2005; Panepinto et al., 2007; Dagley et al., 2011). In addition, mutation of *CCR4* causes hypersensitivity to the echinocandin drug caspofungin in *S. cerevisiae*, *C. albicans* and *C. neoformans* (Markovich et al., 2004; Panepinto et al., 2007; Dagley et al., 2011), suggesting that targeting Ccr4 could be beneficial for combinatorial therapy with the echinocandins. The polyene amphotericin B and the ergosterol biosynthesis inhibitors from the azole family of drugs are the other two classes of antifungals important in the clinical context. An early report in *S. cerevisiae* showed sensitivity of the *ccr4* mutant to the polyene drug nystatin, which binds to membrane ergosterol (Betz et al., 2002). Understanding how Ccr4-NOT mutants in pathogens respond to azoles and polyenes awaits further experimentation.

**Figure 3:**
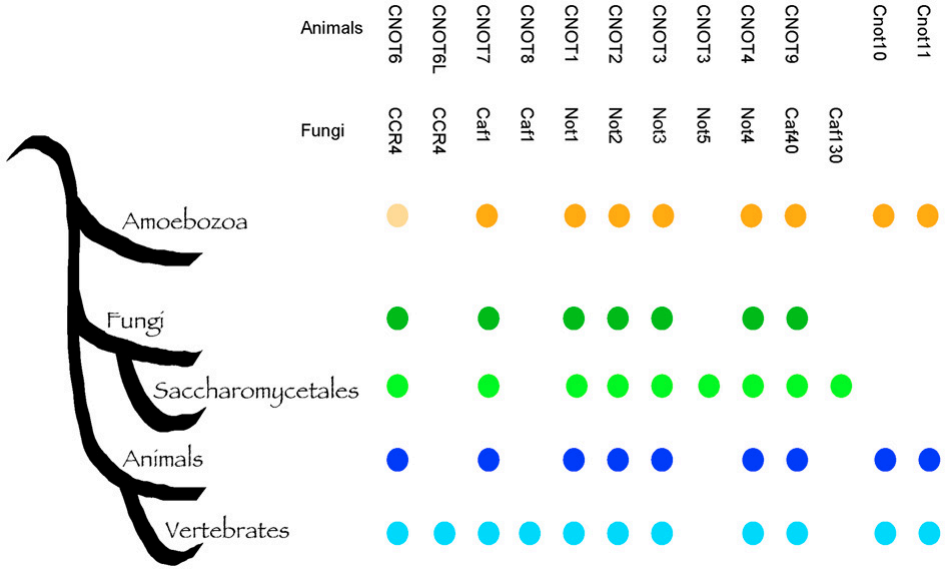
**The evolution of the Ccr4-NOT complex**. A schematic tree indicating the evolution of the indicated groups based on our current understanding; the presence of the respective members is indicated to emphasize fungi-specific gains (Caf130, Not5), as well as loss (CNOT10, CNOT11) due to their prior presence in the *Amoebozoa*. Identifiers for sequences supporting this are shown in Supplemental Table 1. The bright shading of Ccr4 in the *Amoebozoa* indicates the lack of the LRR domain; since this is essential for the binding of Pop2/Caf1 and thus to Not1, this implies that the function of the Ccr4-NOT complex as such could be specific to fungi and animals.

Fungi are eukaryotes and many fundamental cellular pathways are conserved with animals. This close evolutionary relationship poses a problem when fungi become our pathogens, because compounds that inhibit fungal cells can cause host toxicity. The Ccr4-NOT complex is a conserved eukaryotic regulator and potentially, targeting subunits of this complex would result in toxic effect in humans. To address this, we performed a detailed bioinformatic analysis of the conservation of the Ccr4-NOT subunits with animals and within the fungal kingdom. Most the key components of the Ccr4-NOT complex (Ccr4, Caf1/Pop2, Not1, Not2, Not3, Not4, Caf40) were already present before the divergence of fungi and animals (Figure 3), and are readily identified in both groups. However, several fungi-specific differences are noteworthy. The animal CNOT10 and CNOT11 were present in the ancestor, but lost in fungi, whereas Not5 and Caf130 are specific for a sub-group of fungi. While previous analysis realized that Not5 and Caf130 are not conserved between *S. cerevisiae* and animals (Denis and Chen, 2003; Collart and Timmers, 2004), our analysis shows that the acquisition of these two genes is specific for the *Saccharomycetes*, which contain *S. cerevisiae* and *Candida* species, but not other important pathogens such as *Aspergillus* and *Cryptoccous* species (Figure 3; Supplemental Table 1). Caf130 shows no relevant similarity to any other protein and is a highly specific invention (we note that occasionally the fungal Caf130 and the animal CNOT10 are listed as homologs (Miller and Reese, 2012), but there is no relevant sequence conservation between the two proteins). Caf130 is fungal-specific, which makes it an interesting candidate for drug development. The function of Caf130 is not clear at the moment, and the *S. cerevisiae* mutant showed a transcriptional profile similar to the wild type (Azzouz et al., 2009b). However, the *caf130* mutant of *S. cerevisiae* was recently reported to be defective for invasion (Ryan et al., 2012), a phenotype known to affect the virulence potential of *C. albicans*. It is noteworthy that Caf130 is not present outside the *Saccharomycetes*, which would limit its usefulness if the aim were a pan-fungal inhibitor. In contrast to Caf130, Not5 shows considerable sequence similarity to Not3 from animals and fungi, and has arisen from a gene duplication event at the base of the *Saccharomycetes* (Supplemental Figure 1). Our analysis is consistent with previous observations that it is not easily clear whether Not3 or Not5 are more similar to Not3 in organisms with only one copy, as the two genes show similar levels of sequence divergence. Fungi outside the *Saccharomycetes*, such as *Aspergillus* and *Cryptococcus* have only one copy of this gene, which we call *NOT3/5.* While they are easily identifiable as homologs, the sequence conservation of the Not3/Not5 subunit(s) is not very high between organisms, including between fungi and animals (Supplemental Figure 1). Inactivation of *NOT5* has a strong impact on fungal fitness, and in *C. albicans* the mutant has a cell wall and a virulence defect (Cheng et al., 2005). Collectively, the sequence divergence and roles in virulence suggest that the Not5/Not3 proteins could be targets of interest in the context of antifungal therapy.

**Figure 4:**
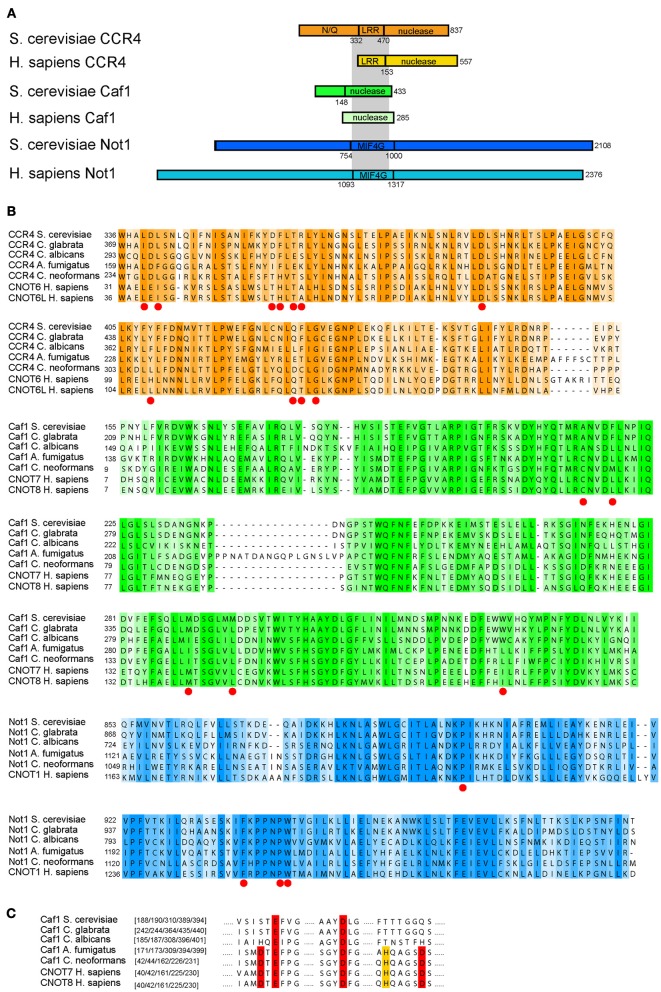
**Comparison of Ccr4-Caf1-Not1 interaction domains between humans and pathogenic fungi**. **(A)** A schematic overview of the Ccr4-Caf1-Not1 interactions based on *S. cerevisiae*, illustrating the interactions between the Ccr4 LRR domain, Caf1, and the Not1 MIF4G fold domain as reported in Basquin et al. (2012) and Petit et al. (2012); the gray shading indicates the interactions. **(B)** Alignment of the corresponding regions of Ccr4, Caf1, and Not1 in yeast and several fungal pathogens compared to the human homologs. The sites described to be responsible for the function and/or protein-protein interactions in yeast (Clark et al., 2004; Basquin et al., 2012) are indicated by red dots. Color shades represent sequence conservation with a 20% cutoff value as implemented in JalView (Clamp et al., 2004). **(C)** The DEDD motif of Caf1, as well as the histidine that aids in formation of the cofactor binding pocket (Jonstrup et al., 2007; Horiuchi et al., 2009), are highlighted in red or yellow, respectively. This illustrates the loss of these residues in the *Saccharomycetes*, but not in other fungal groups. The sequences represent a subset from a full alignment of all sequences. The amino acids are indicated at the start of each block in **(B)**, and the highlighted amino acid residue numbers are given in brackets for each sequence in **(C)**. All alignments are based on the full sequence selection of fungi and animal sequences as given in Supplemental Table 1, and were aligned with muscle (version 3.8.31) (Edgar, 2004), using default settings. Shown sequences were subsequently selected and only-gap sites removed.

We next investigated the level of sequence conservation for the deadenylase subunits between humans and the fungal pathogens (*Cryptococcus*, *Candida*, and *Aspergillus*; Figure 4). Despite significant differences in length, the Ccr4/Caf1/Not1 interacting domains are conserved between the proteins of fungi and humans (Figures 4A,B) (Basquin et al., 2012). We focused on the sequence regions known to hold the essential sites for the interactions between Ccr4-Caf1 and Caf1-Not1, as determined in a recent structural study (Basquin et al., 2012). The Caf1-Not1 interaction anchors Ccr4 into the Ccr4-NOT complex, and the interactions between these three subunits are essential for deadenylation and growth of *S. cerevisiae* (Basquin et al., 2012). The high level of conservation of the sites in Ccr4, Pop2/Caf1, and Not1 required for the interactions indicate that functionality of these regions is likely conserved for fungi and animals (Figure 4B). Despite the high similarity, there is a level of variation in the sequences, which could prove beneficial to design compounds to specifically target the fungal complex. We note that loss of the catalytic deadenylase activity of Caf1/Pop2 and the DEDD motif, which is archetypical for this family of deadenylases, is true only for the *Saccharomycetes* (which include *S. cerevisiae* and the *Candida* species), but not the other fungal pathogens (Figure 4C).

The presence of CNOT10 in organisms other than animals (Figure 3) indicates its loss in fungi and potential functional replacement with Caf130, for which no similar proteins outside the *Saccharomycetes* could be found. This might go in accordance with the differences observed in Not1; whilst the C-terminus, the binding site of Caf1, Ccr4, and Not2-5 (Basquin et al., 2012), is relatively conserved, the N-terminus - which harbors the Caf40 and CNOT10 /CNOT11, and possibly also the Caf130, binding sites (Nasertorabi et al., 2011; Bawankar et al., 2013) - is reduced in fungi compared to animals (Figure 4A). In the *Candida* group, the proteins lack approximately 180 amino acids at the N-terminal part compared to *S. cerevisiae* (Supplemental Figure 2 shows an alignment of Not1 sequences from *S. cerevisiae*, representative fungal pathogens and humans). The Not1 MIF4G region in Not1 contains several sites known to be essential for binding of Caf1 (to which Ccr4 binds) in both fungi and animals (Basquin et al., 2012; Petit et al., 2012) (Figure 4A).

In conclusion, while the Ccr4-NOT complex is generally conserved between yeast and humans, some subunits are fungal-specific, and some sequence variation can be observed in the conserved subunits. These observations suggest that this complex might be explored in the context of antifungal therapy. It is noteworthy that developing antifungal drugs against targets conserved with humans has been done successfully: the target of the commonly used azole drugs, sterol 14 α-demethlyase, is an evolutionarily conserved enzyme required for ergosterol/cholesterol synthesis. However, the inhibition of the fungal enzyme by azoles is higher than inhibition of the human homolog (Parker et al., 2008; Warrilow et al., 2013). It might also be useful to think about Ccr4-NOT in the context of combinatorial therapy with conventional antifungal drugs, such as the echinocandins. One can imagine that the deadenylase activities of Ccr4 could be targeted, and the levels of inhibition adjusted so not to cause adverse effects on human cells, but rather specifically kill fungi in combination with other, fungal-specific drugs.

## Conclusions and outlook

Ccr4-NOT has been implicated in the ability of *C. albicans* and *C. neoformans* to cause disease, despite fundamental differences in the pathogenic processes of these two human pathogens. For example, *C. albicans* is a human commensal and the temperature of the human host is not sensed as a stressor. In contrast, *C. neoformans* is an environmental fungus, and the shift from environmental temperature to human core body temperature is sensed as a stressor and involves reprogramming of mRNA pools. Both *C. albicans* and *C. neoformans* encounter macrophages. *C. neoformans* has evolved the ability to survive and even proliferate within the macrophage, whereas *C. albicans* utilizes the yeast-to-hyphae morphogenetic transition to escape the phagocyte. Though the role of Ccr4 in the survival of *C. neoformans* or *C. albicans* in macrophages has not been investigated, the stress sensitivity of the *C. neoformans* mutant and the lack of morphogenetic capacity of the *C. albicans* mutant predict that the macrophage-pathogen interaction would lead to elimination of the pathogen. This illustrates how the pleoiotropic phenotypes of the *ccr4-not* mutants might be beneficial in targeting diverse pathogens, in addition to inactivating several processes important for virulence. Further studies of Ccr4-NOT function in *C. albicans* and *C. neoformans*, as well as extension to other pathogenic species, such as the molds and the dimorphs, are likely to be informative for understanding fungal pathogenesis and provide further insight how this complex or a pathway that it regulates could be targeted for improving treatments.

## Author contributions

Eva Heinz performed the bioinformatic analysis. John C. Panepinto, Eva Heinz, and Ana Traven wrote the manuscript.

### Conflict of interest statement

The authors declare that the research was conducted in the absence of any commercial or financial relationships that could be construed as a potential conflict of interest.

## Supplementary material

Supplemental Figure 1 **The relationship between Not3, Not5, and CNOT3 with respect to sequence divergence and phylogenetics**. **(A)** Both Not3 and Not5 are present only in the *Saccharomycetes*. Sequence accession numbers are given in Supplemental Table 1. The alignment was performed by muscle (version 3.8.31) (Edgar, 2004) using default settings. The color shades represent sequence conservation with a 20% cutoff value, and the level of conservation is shown, as implemented in JalView (Clamp et al., 2004). **(B)** Phylogenetic analysis reveals that the Not3 and Not5 are products of a gene duplication (the duplication event is indicated by a red dot), and show similar levels of sequence divergence, supporting the idea that it is not clear whether the *Saccharomycetes* Not3 or Not5 is more similar to the single-copy Not3 (CNOT3). The sequence accession numbers are given in Supplemental Table 1. The sequences were aligned with muscle (version 3.8.31) (Edgar, 2004) using default conditions, and divergent regions were removed using trimal (1.2rev59) (Capella-Gutierrez et al., 2009) with the “-automated1” setting. The tree was calculated in MrBayes (version 3.2.1) (Ronquist and Huelsenbeck, 2003) using the amino acid mixture model with 10.000.000 generations, with 25% discarded as burn-in during the consensus tree calculation; support values shown are posterior probabilities.

Supplemental Figure 2 **Divergence of Not1 between fungi and humans**. Not1 shows different profiles of conservation, which goes in accordance with the binding domains of conserved (Ccr4, Caf1) and divergent (Caf40, Caf130 vs. CNOT10) binding partners. The alignment was performed by muscle (version 3.8.31) (Edgar, 2004) using default settings, color shades represent sequence conservation with a 20% cutoff value as implemented in JalView (Clamp et al., 2004); accession numbers of all sequences are given in Supplemental Table 1.

Supplemental Table 1 **Table accessions**. Accession numbers and sequence data were retrieved from the yeast gene order browser (http://ygob.ucd.ie/) (Byrne and Wolfe, 2005), the *Candida* gene order browser (http://cgob.ucd.ie/) (Maguire et al., 2013) or the NCBI RefSeq database.
