# Supplementary material for: The cellular roles of Ccr4-NOT in model and pathogenic fungi—implications for fungal virulence
Source: Front Genet. 2013 Dec 20;4:302. doi: 10.3389/fgene.2013.00302 (PMC3868889; doi:10.3389/fgene.2013.00302)
Supplement: Supplemental Figure 1 — The relationship between Not3, Not5, and CNOT3 with respect to sequence divergence and phylogenetics. (A) Both Not3 and Not5 are present only in the Saccharomycetes. Sequence accession numbers are given in Supplemental Table 1. The alignment was performed by muscle (version 3.8.31) (Edgar, 2004) using default settings. The color shades represent sequence conservation with a 20% cutoff value, and the level of conservation is shown, as implemented in JalView (Clamp et al., 2004). (B) Phylogenetic analysis reveals that the Not3 and Not5 are products of a gene duplication (the duplication event is indicated by a red dot), and show similar levels of sequence divergence, supporting the idea that it is not clear whether the Saccharomycetes Not3 or Not5 is more similar to the single-copy Not3 (CNOT3). The sequence accession numbers are given in Supplemental Table 1. The sequences were aligned with muscle (version 3.8.31) (Edgar, 2004) using default conditions, and divergent regions were removed using trimal (1.2rev59) (Capella-Gutierrez et al., 2009) with the “-automated1” setting. The tree was calculated in MrBayes (version 3.2.1) (Ronquist and Huelsenbeck, 2003) using the amino acid mixture model with 10.000.000 generations, with 25% discarded as burn-in during the consensus tree calculation; support values shown are posterior probabilities. [file Presentation1.PDF]

(A)

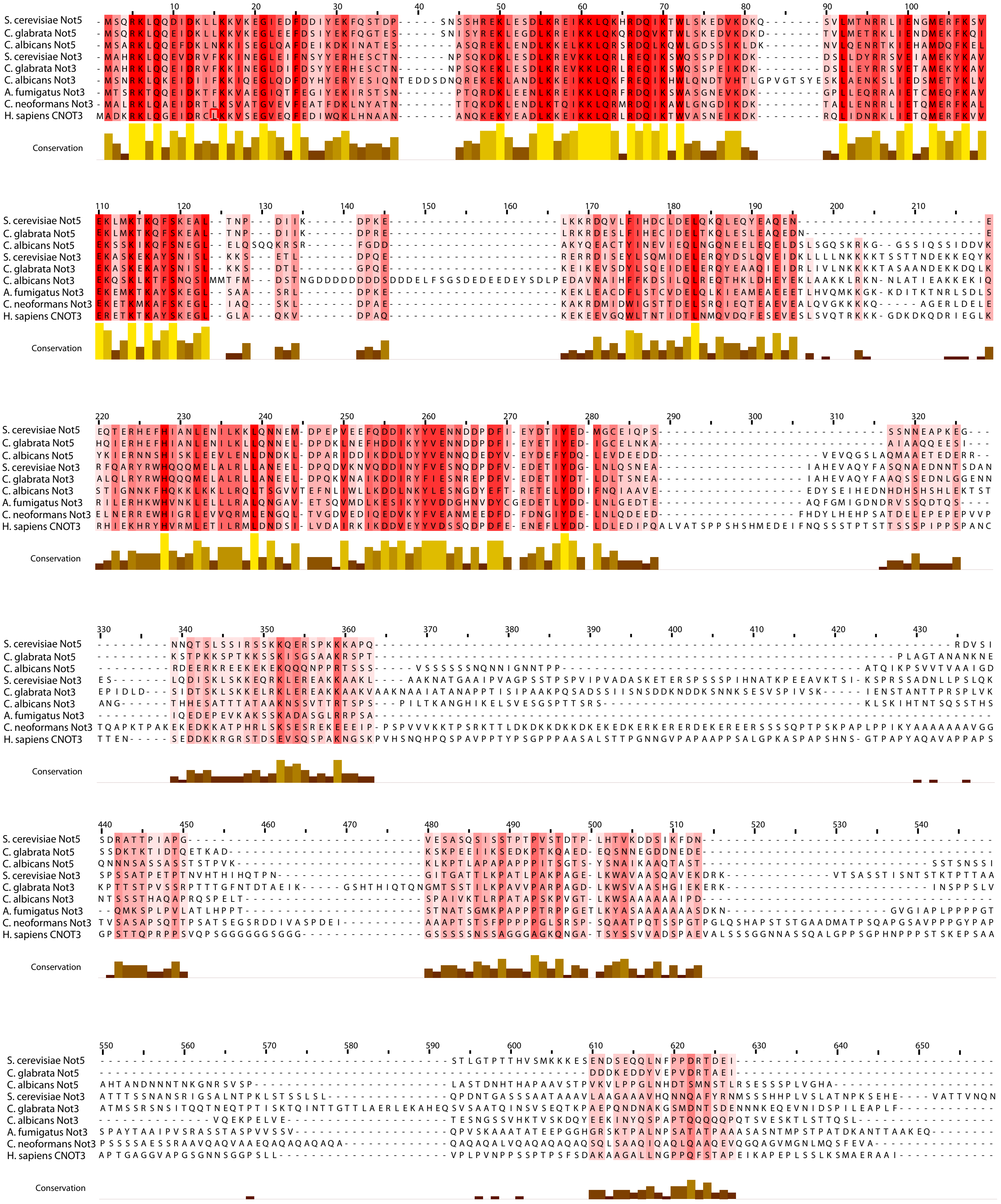
S. cerevisiae Not5
C. glabrata Not5
C. albicans Not5
S. cerevisiae Not3
C. glabrata Not3
C. albicans Not3
A. fumigatus Not3
C. neoformans Not3
H. sapiens CNOT3
Conservation

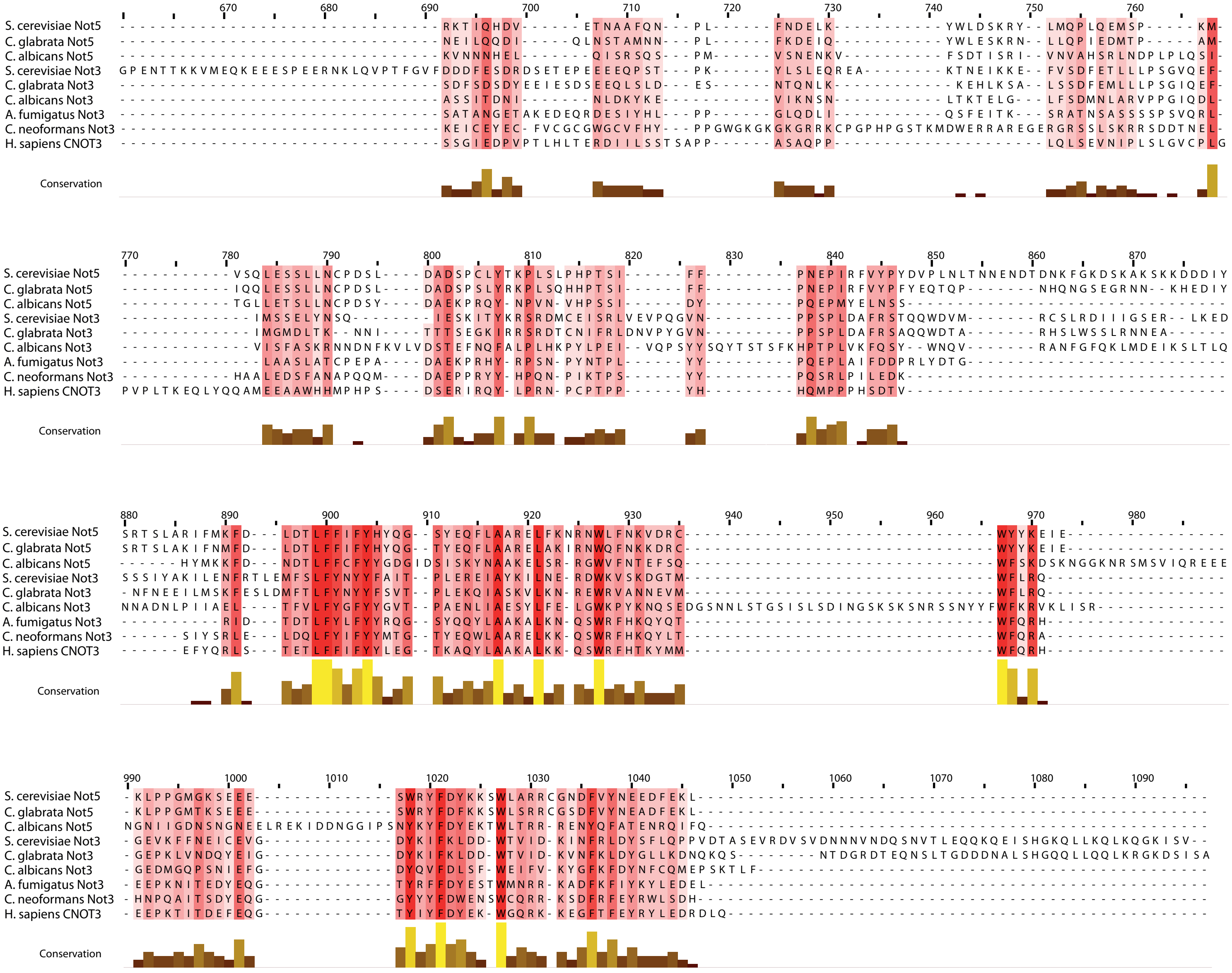
S. cerevisiae Not5
C. glabrata Not5
C. albicans Not5
S. cerevisiae Not3
C. glabrata Not3
C. albicans Not3
A. fumigatus Not3
C. neoformans Not3
H. sapiens CNOT3
Conservation

(B)

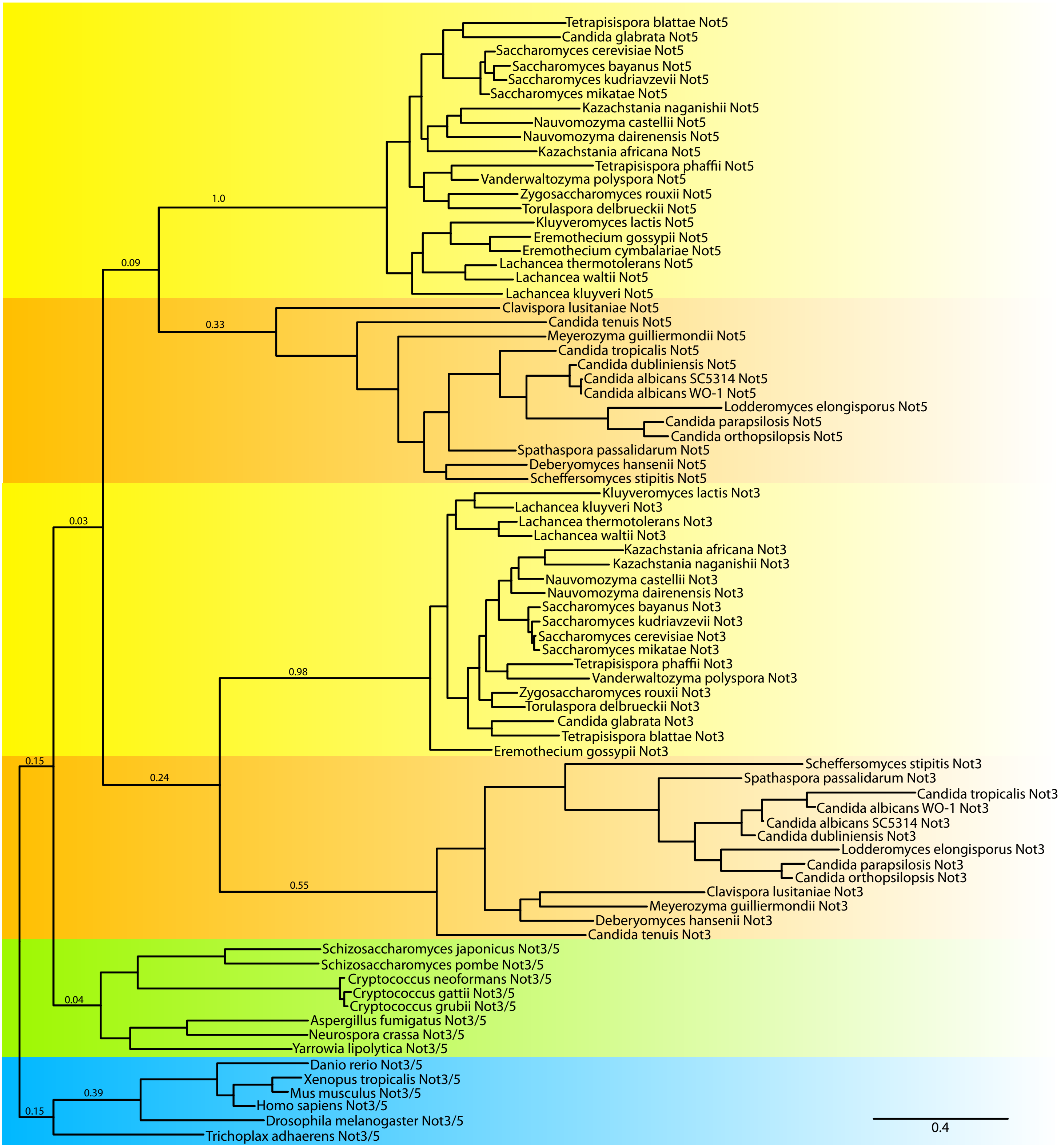
Tetrapisispora blattae Not5
Candida glabrata Not5
Saccharomyces cerevisiae Not5
Saccharomyces bayanus Not5
Saccharomyces kudriavzevii Not5
Saccharomyces mikatae Not5
Kazachstania naganishii Not5
Nauvomozyma castellii Not5
Nauvomozyma dairenensis Not5
Kazachstania africana Not5
Tetrapisispora phaffii Not5
Vanderwaltozyma polyspora Not5
Zygosaccharomyces rouxii Not5
Torulaspora delbrueckii Not5
Kluyveromyces lactis Not5
Eremothecium gossypii Not5
Eremothecium cymbalariae Not5
Lachancea thermotolerans Not5
Lachancea waltii Not5
Lachancea kluyveri Not5
1.0
0.09
Clavispora lusitaniae Not5
Candida tenuis Not5
Meyerozyma guilliermondii Not5
Candida tropicalis Not5
Candida dubliniensis Not5
Candida albicans SC5314 Not5
Candida albicans WO-1 Not5
Lodderomyces elongisporus Not5
Candida parapsilosis Not5
Candida orthopsilopsis Not5
Spathaspora passalidarum Not5
Deberyomyces hansenii Not5
Scheffersomyces stipitis Not5
0.33
0.03
Kluyveromyces lactis Not3
Lachancea kluyveri Not3
Lachancea thermotolerans Not3
Lachancea waltii Not3
Kazachstania africana Not3
Kazachstania naganishii Not3
Nauvomozyma castellii Not3
Nauvomozyma dairenensis Not3
Saccharomyces bayanus Not3
Saccharomyces kudriavzevii Not3
Saccharomyces cerevisiae Not3
Saccharomyces mikatae Not3
Tetrapisispora phaffii Not3
Vanderwaltozyma polyspora Not3
Zygosaccharomyces rouxii Not3
Torulaspora delbrueckii Not3
Candida glabrata Not3
Tetrapisispora blattae Not3
Eremothecium gossypii Not3
0.98
0.15
0.24
Scheffersomyces stipitis Not3
Spathaspora passalidarum Not3
Candida tropicalis Not3
Candida albicans WO-1 Not3
Candida albicans SC5314 Not3
Candida dubliniensis Not3
Lodderomyces elongisporus Not3
Candida parapsilosis Not3
Candida orthopsilopsis Not3
Clavispora lusitaniae Not3
Meyerozyma guilliermondii Not3
Deberyomyces hansenii Not3
Candida tenuis Not3
0.55
Schizosaccharomyces japonicus Not3/5
Schizosaccharomyces pombe Not3/5
Cryptococcus neoformans Not3/5
Cryptococcus gattii Not3/5
Cryptococcus grubii Not3/5
Aspergillus fumigatus Not3/5
Neurospora crassa Not3/5
Yarrowia lipolytica Not3/5
0.04
Danio rerio Not3/5
Xenopus tropicalis Not3/5
Mus musculus Not3/5
Homo sapiens Not3/5
Drosophila melanogaster Not3/5
Trichoplax adhaerens Not3/5
0.39
0.15
0.4
