# Supplementary material for: The cellular roles of Ccr4-NOT in model and pathogenic fungi—implications for fungal virulence
Source: Front Genet. 2013 Dec 20;4:302. doi: 10.3389/fgene.2013.00302 (PMC3868889; doi:10.3389/fgene.2013.00302)
Supplement: Supplemental Figure 2 — Divergence of Not1 between fungi and humans. Not1 shows different profiles of conservation, which goes in accordance with the binding domains of conserved (Ccr4, Caf1) and divergent (Caf40, Caf130 vs. CNOT10) binding partners. The alignment was performed by muscle (version 3.8.31) (Edgar, 2004) using default settings, color shades represent sequence conservation with a 20% cutoff value as implemented in JalView (Clamp et al., 2004); accession numbers of all sequences are given in Supplemental Table 1. [file Presentation2.PDF]

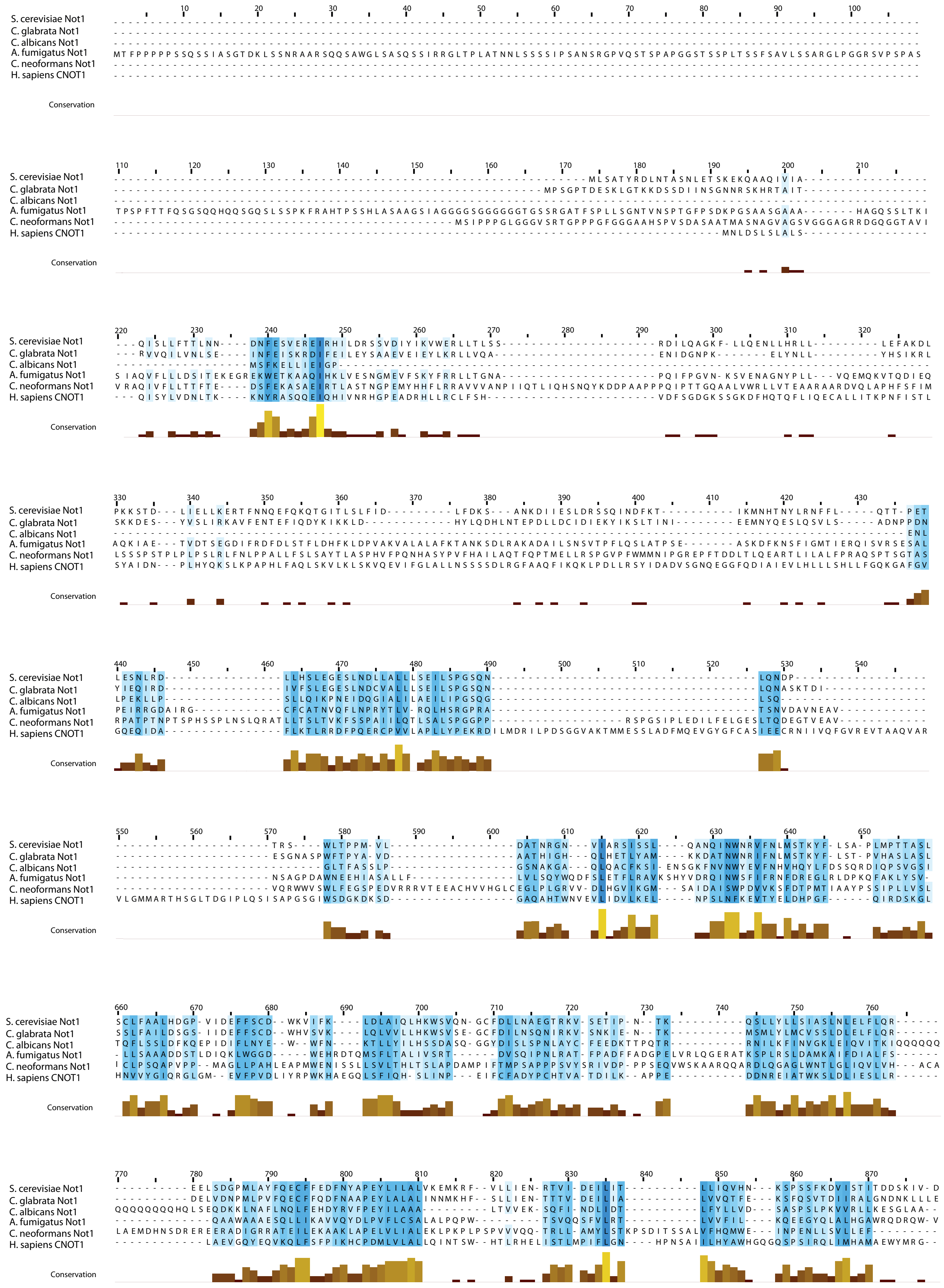
S. cerevisiae Not1
C. glabrata Not1
C. albicans Not1
A. fumigatus Not1
C. neoformans Not1
H. sapiens CNOT1
Conservation

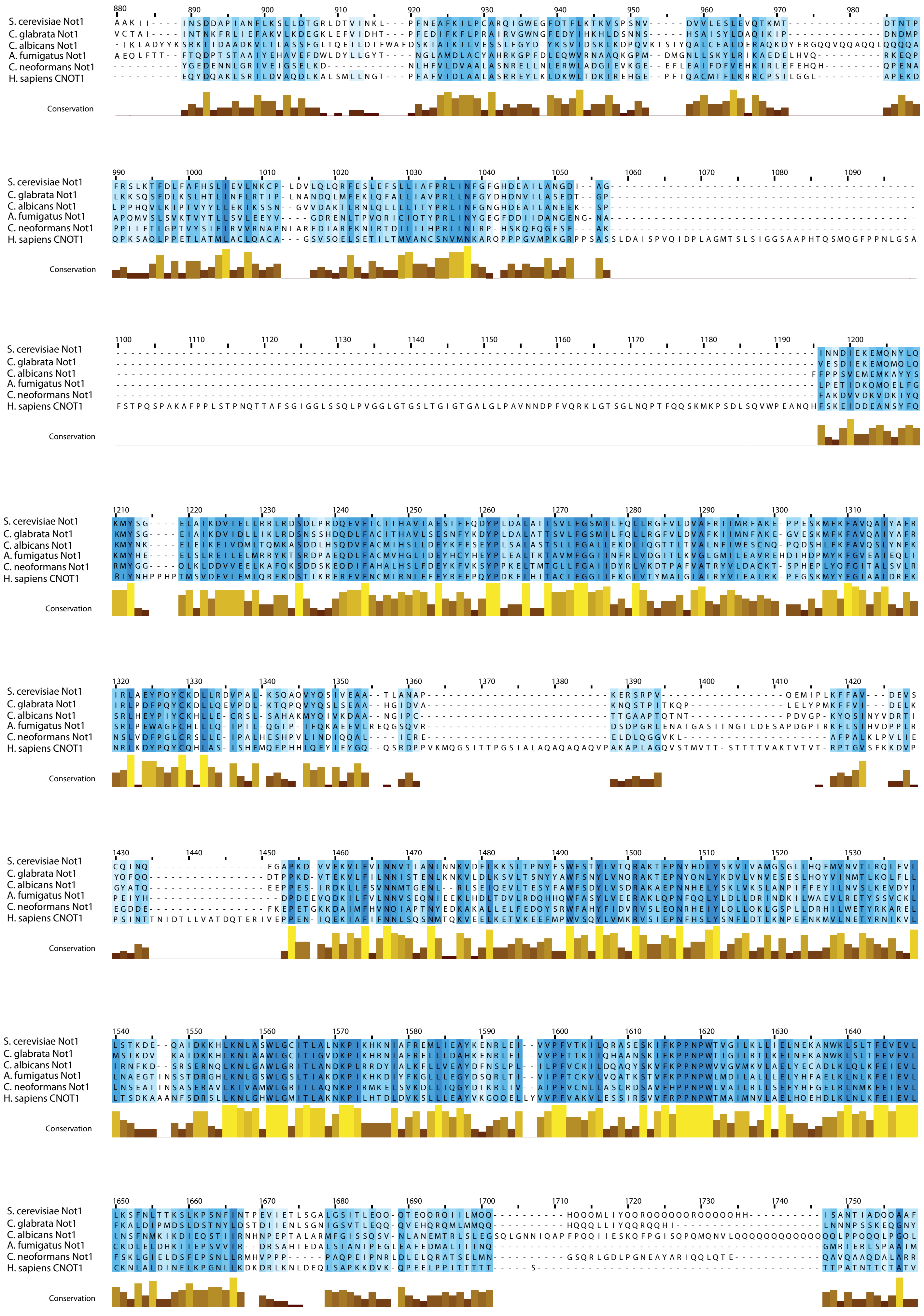
S. cerevisiae Not1
C. glabrata Not1
C. albicans Not1
A. fumigatus Not1
C. neoformans Not1
H. sapiens CNOT1
Conservation

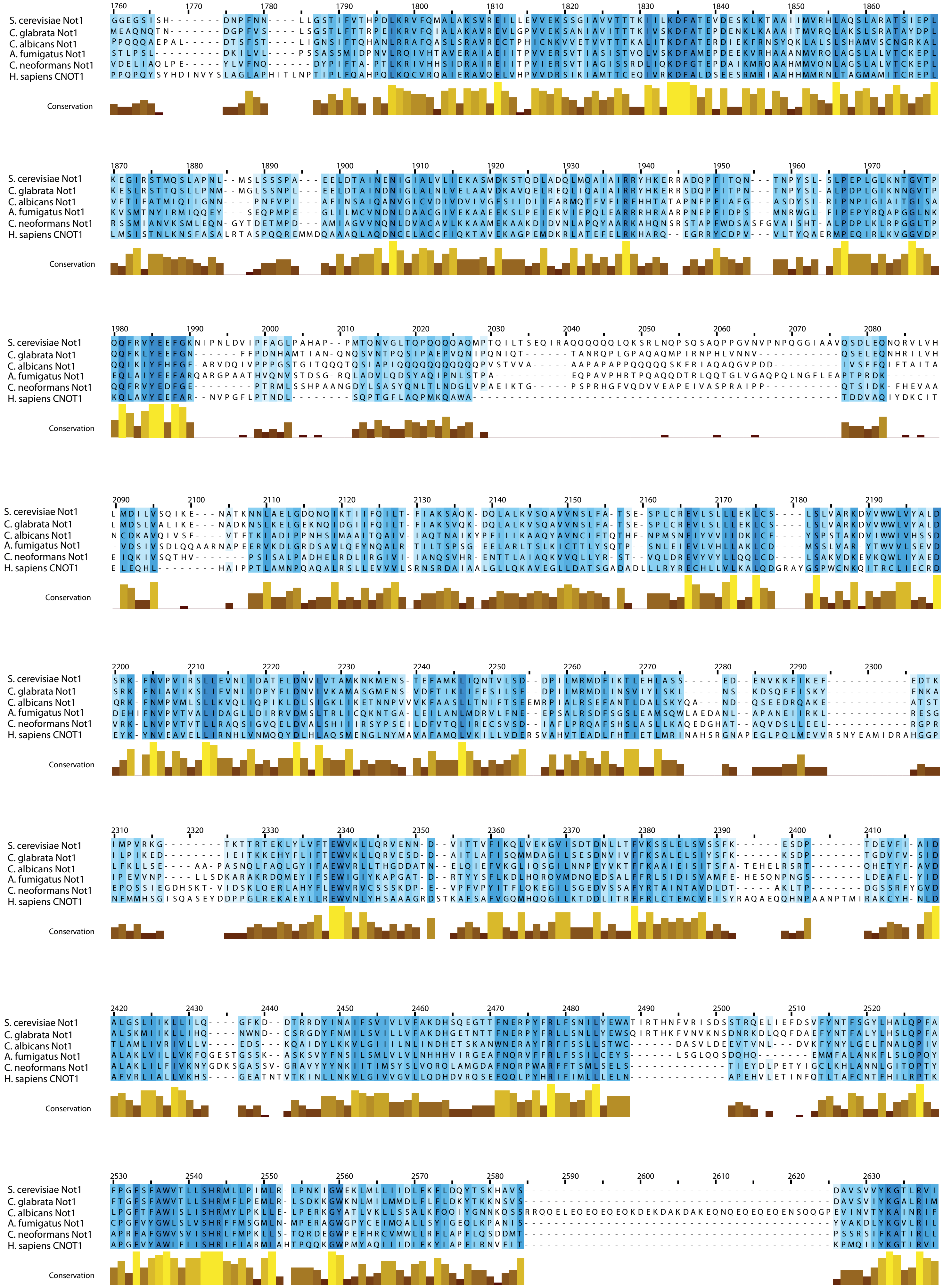
S. cerevisiae Not1
C. glabrata Not1
C. albicans Not1
A. fumigatus Not1
C. neoformans Not1
H. sapiens CNOT1
Conservation

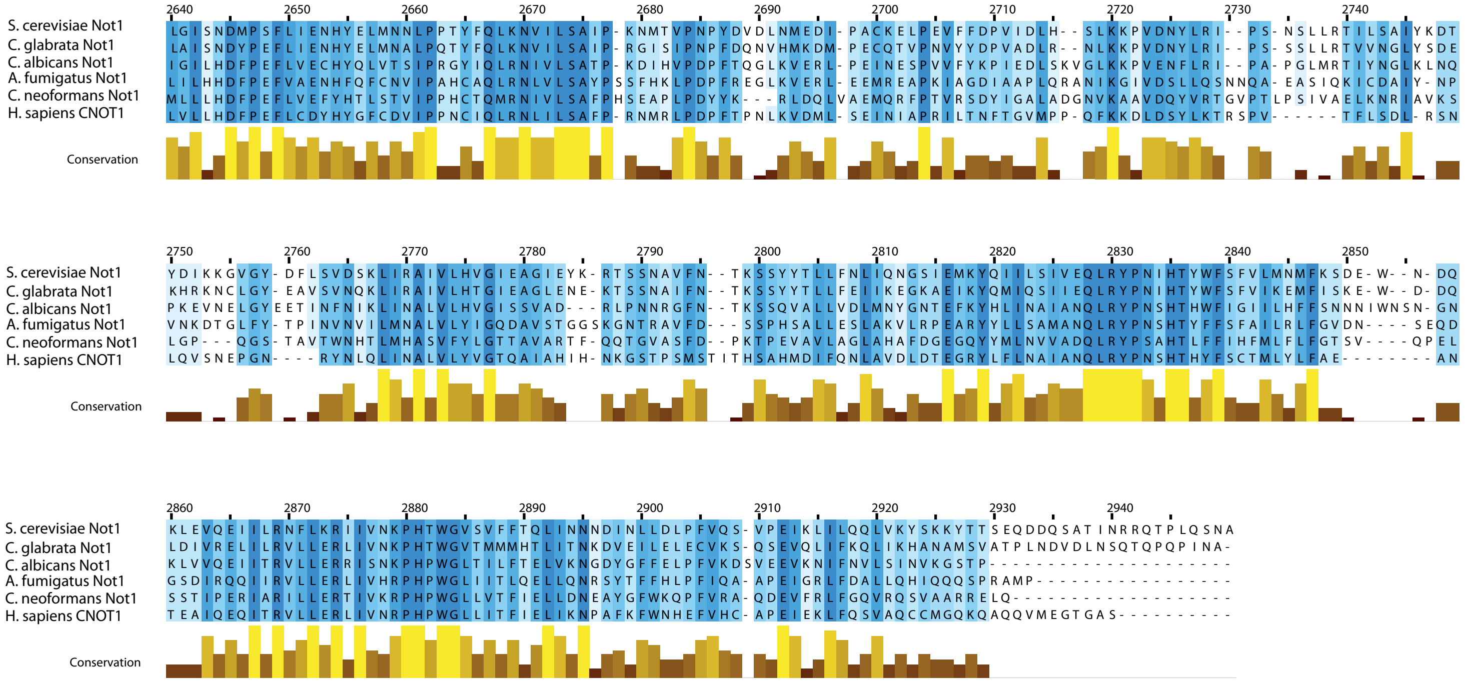
2640 2650 2660 2670 2680 2690 2700 2710 2720 2730 2740
S. cerevisiae Not1
C. glabrata Not1
C. albicans Not1
A. fumigatus Not1
C. neoformans Not1
H. sapiens CNOT1
Conservation
2750 2760 2770 2780 2790 2800 2810 2820 2830 2840 2850
S. cerevisiae Not1
C. glabrata Not1
C. albicans Not1
A. fumigatus Not1
C. neoformans Not1
H. sapiens CNOT1
Conservation
2860 2870 2880 2890 2900 2910 2920 2930 2940
S. cerevisiae Not1
C. glabrata Not1
C. albicans Not1
A. fumigatus Not1
C. neoformans Not1
H. sapiens CNOT1
Conservation
